# Proteomic profiles and the function of RBP4 in endometrium during embryo implantation phases in pigs

**DOI:** 10.1186/s12864-023-09278-5

**Published:** 2023-04-13

**Authors:** Yueying Wang, Songyi Xue, Qiaorui Liu, Dengying Gao, Renwu Hua, Minggang Lei

**Affiliations:** 1 Department of Reproductive Medicine, Jining No.1 People’s Hospital, Jining, 272000 China; 2 Key Laboratory of Agricultural Animal Genetics, Breeding, and Reproduction of the Ministry of Education and Key Laboratory of Swine Genetics and Breeding of the Ministry of Agriculture, College of Animal Science and Technology, Huazhong Agricultural University, Wuhan, 430000 China; 3 Shenzhen Key Laboratory of Fertility Regulation, Center of Assisted Reproduction and Embryology, The University of Hong Kong-Shenzhen Hospital, Shenzhen, 518053 China; 4 Center for Energy Metabolism and Reproduction, Shenzhen Institute of Advanced Technology, Chinese Academy of Sciences, Shenzhen, 518055 China

**Keywords:** Endometrium, Implantation, Proteomic profiles, RBP4

## Abstract

**Background:**

Endometrial receptivity plays a vital role in the success of embryo implantation. However, the temporal proteomic profile of porcine endometrium during embryo implantation is still unclear.

**Results:**

In this study, the abundance of proteins in endometrium on days 9, 10, 11, 12, 13, 14, 15 and 18 of pregnancy (D9, 10, 11, 12, 13, 14, 15 and 18) was profiled via iTRAQ technology. The results showed that 25, 55, 103, 91, 100, 120, 149 proteins were up-regulated, and 24, 70, 169, 159, 164, 161, 198 proteins were down-regulated in porcine endometrium on D10, 11, 12, 13, 14, 15 and 18 compared with that on D9, respectively. Among these differentially abundance proteins (DAPs), Multiple Reaction Monitoring (MRM) results indicated that S100A9, S100A12, HRG and IFI6 were differentially abundance in endometrial during embryo implantation period. Bioinformatics analysis showed that the proteins differentially expressed in the 7 comparisons were involved in important processes and pathways related to immunization, endometrial remodeling, which have a vital effect on embryonic implantation.

**Conclusion:**

Our results reveal that retinol binding protein 4 (RBP4) could regulate the cell proliferation, migration and apoptosis of endometrial epithelial cells and endometrial stromal cells to affect embryo implantation. This research also provides resources for studies of proteins in endometrium during early pregnancy.

**Supplementary Information:**

The online version contains supplementary material available at 10.1186/s12864-023-09278-5.

## Introduction

The embryo implantation period has been recognized as a vital stage that influences the litter size of sows [1, 2]. Endometrial receptivity, the development of embryos and synchronous communication between the endometrium and embryos have an important impact on the process of embryo implantation [3, 4]. From Day (D) 9 of pregnancy to D12 of pregnancy, the blastocyst changes rapidly from spherical (1–2 mm) to ovoid (4–5 mm) and tubular (> 10 mm) and finally elongates into a filamentous form within 2–3 h (> 100 mm) [5, 6]. Embryos move in the uterine cavity and determine their attachment position in the uterus on D12 and then reach the surface of the endometrium on D13 [7, 8]. The apical surface of the endometrial epithelium forms pinopods that become enclosed by caps of trophectoderm cells through D13 and D14 [9]. The function of the maternal–fetal interface changes from histotrophic to histotrophic and hemotrophic nutrient transport after D15, and implantation is usually completed between D18 and D24 of pregnancy [10]. Despite our increased knowledge on the role of the endometrium in embryo implantation, only limited information is available about the molecular regulation in the endometrium during the embryo implantation period.

Previous studies have focused on the gene expression of the endometrium by transcriptome sequencing during the embryo implantation period [11–15]. Due to posttranscriptional and posttranslational regulation, the transcription results cannot fully represent the changes in the protein during porcine embryo implantation [16]. Proteomic approaches have also been used to study differentially expressed proteins in the endometrium based on the two-dimensional gel electrophoresis (2DE) method during early pregnancy in pigs [17, 18]. However, isobaric tags for relative and absolute quantification (iTRAQ), enabling the identification and quantification of lower abundance proteins, has not yet been employed to analyze the proteomic profile of the endometrium during porcine embryo implantation. 2DE is a top-down approach that quantifies the differentially abundant proteins at the protein level before identifying the protein by LC–MS/MS, while the iTRAQ method is a bottom-up approach in which the whole proteome is first digested with trypsin, and the generated peptides are separated by chromatography and identified and measured by mass spectrometry.

In this study, the abundance of proteins in the endometrium during embryo implantation was comprehensively profiled using iTRAQ technology in Yorkshire (YK) pigs. Moreover, we identified a candidate protein that may play a vital role in embryo implantation. The principal goal of the research was to provide a resource for studies on the temporal abundance profile of proteins during embryo implantation.

## Result

### iTRAQ analysis reveals changes in protein abundance in porcine endometrium on different gestation days (D9-15 and 18)

**Fig. 1:**
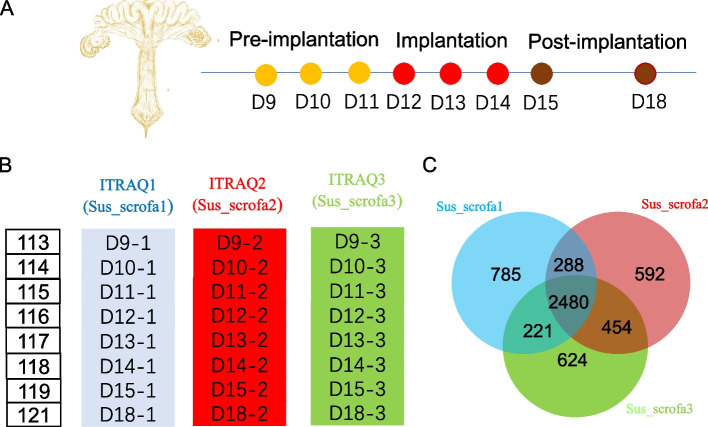
The overview of iTRAQ experiment design. **A** Total proteins were extracted from porcine endometrium on pregnancy of days (D) 9, 10, 11, 12, 13, 14, 15 and 18 (*n* = 3). **B** Three independent 8-plex iTRAQ experiments were performed with the internal reference (the endometrium on D9) on each of the experiments. **C** Venn Diagram indicated that 2480 commonly identified proteins among the three iTRAQ experiments

An iTRAQ-based quantitative proteomic method was used to obtain the proteomic profile of the porcine endometrium on the following gestation days: D9-15 and 18 (Fig. 1A). The porcine endometrium on D9 was used as the internal reference for three independent iTRAQ experiments (Fig. 1B). A total of 3,774, 3,814 and 3,779 proteins were detected in each of the three iTRAQ experiments. Among these proteins, 2,480 proteins were detected in all three iTRAQ experiments. Then, we used these proteins to characterize the proteomic profiles of the porcine endometrium on different gestation days and carried out different statistical comparisons (Fig. 1C).

**Fig. 2:**
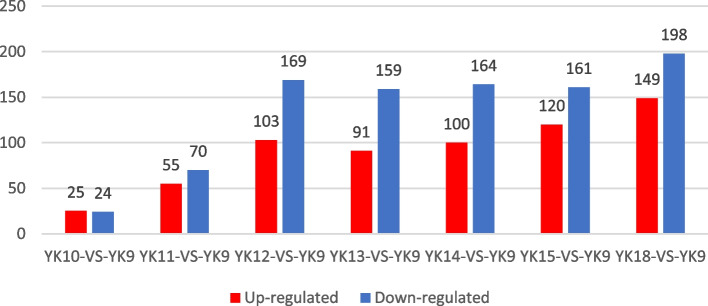
The differently expressed proteins in different comparison groups. YK: Days of pregnancy for Yorkshire pigs. Red shows up-regulated and blue shows down-regulated

The proteins with |Fold change|> 1.2 and Q-value < 0.05 in each comparison group were defined as differentially abundance proteins (DAPs). The results showed that 25, 55, 103, 91, 100, 120, and 149 proteins were upregulated, and 24, 70, 169, 159, 164, 161, and 198 proteins were downregulated in porcine endometrium on D10, 11, 12, 13, 14, 15 and 18 compared with those on D9, respectively (Fig. 2).

**Fig. 3:**
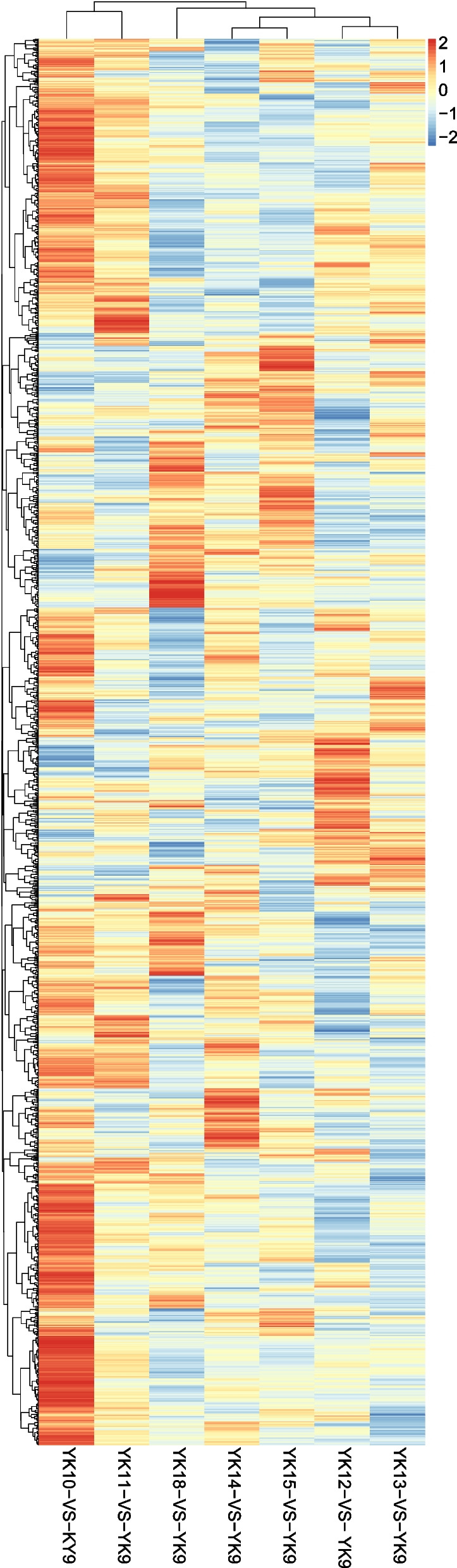
Hierarchical cluster analysis of different abundance expression. YK: Days of pregnancy for Yorkshire pigs. Red shows higher fold change and blue shows lower fold change

To explore the differences in the protein abundance pattern of the porcine endometrium during different phases of embryo implantation, hierarchical cluster analysis (HCA) was performed to cluster all proteins and their abundance profiles in different groups (Fig. 3) (Additional file 1: Table S1). The results indicated that the abundance profiles of proteins in the YK10 vs. YK9 and YK11 vs. YK9 comparison groups were directly clustered together, and the YK12 vs. YK9 and YK13 vs. YK9 comparison groups were also directly clustered together. Additionally, the abundance profiles of the YK14 vs. YK9 and YK15 vs. YK9 comparison groups were similar.

### Time-series expression analysis

**Fig. 4:**
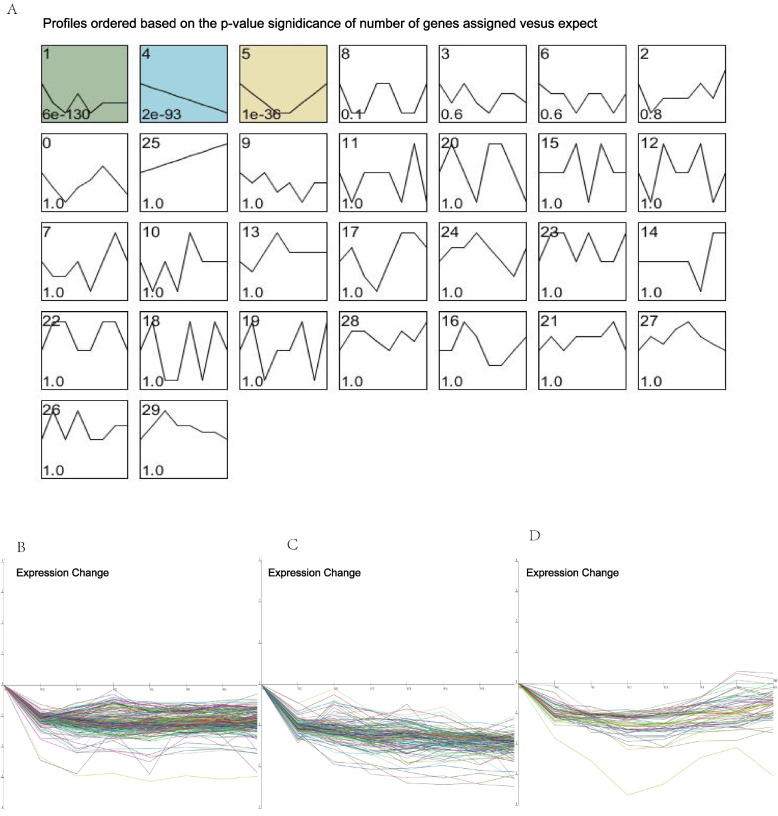
STEM clustering based on different abundance protein. **A** Each square represents a kind of expression profile and colored squares mean statistical significance. The upper-left number in the box indicates the order of profile. The bottom-left number indicates the *P*-value. Polyline in the box presents the changing pattern of protein expression levels. **B** **C** **D** Detailed descriptions of protein expression patterns for “profile 1”, “profile 4” and “profile 5”, respectively

The short time-series expression miner (STEM) was used to investigate the pattern of differentially abundant protein (DAP) expression during the 8 stages (Additional file 2: Table S2). The typical abundance patterns of proteins (*P* < 0.05) were profile 1, profile 4 and profile 5 (Fig. 4A). The expression patterns of proteins in profile 1 and profile 4 were characterized by decreased levels from D9 to D18 (Fig. 4B and Fig. 4C). The abundance levels of proteins in profile 5 reached a minimum on D12 and D13 and significantly increased thereafter, but there was no significant change between the expression levels of proteins in profile 5 on D9 and D18 (Fig. 4D).

### MRM method validation

The multiple reaction monitoring (MRM) method was carried out on 8 chosen differentially expressed proteins to verify the reliability of the iTRAQ results. All Pearson correlation coefficients between the MRM data and iTRAQ data were > 0.65, indicating that the expression patterns of protein determined by MRM were generally consistent with those obtained by iTRAQ (Table 1).

**Table 1:** Validation of iTRAQ results by using MRM method

Protein ID		YK9vs YK9	YK10 vs YK9	YK11 vs YK9	YK12 vs YK9	YK13 vs YK9	YK14 vs YK9	YK15 vs YK9	YK18 vs YK9	Correlation
sp|P80310|S10AC_PIG (S100A12)	iTRAQ	1.00	1.51	1.33	1.99	1.56	1.43	2.38	1.67	0.92
	MRM	1.00	4.04	2.85	7.00	2.75	1.23	9.54	2.73	
tr|F1SFI5|F1SFI5_PIG (HRG)	iTRAQ	1.00	0.90	1.13	1.57	1.45	1.16	1.54	1.20	0.91
	MRM	1.00	1.07	1.95	2.38	2.51	1.94	2.60	2.05	
tr|D3Y264|D3Y264_PIG (APOC2)	iTRAQ	1.00	0.97	1.27	1.16	1.28	1.24	1.21	1.30	0.91
	MRM	1.00	1.44	3.61	2.65	3.84	2.73	2.15	4.00	
sp|A5GFY8|SERA_PIG (PHGDH)	iTRAQ	1.00	1.03	0.83	0.75	0.68	0.63	0.59	0.55	0.91
	MRM	1.00	0.99	0.43	0.34	0.46	0.31	0.36	0.26	
sp|P27917|APOC3_PIG (APOC3)	iTRAQ	1.00	0.68	0.96	1.23	1.57	1.44	1.24	1.70	0.88
	MRM	1.00	0.85	2.10	1.91	2.85	2.04	1.92	3.43	
tr|C3S7K6|C3S7K6_PIG (S100A9)	iTRAQ	1.00	1.29	1.77	2.80	3.02	1.60	2.67	1.70	0.78
	MRM	1.00	3.72	4.22	42.17	13.82	4.93	28.09	5.33	
tr|Q6IED5|Q6IED5_PIG (IFI6)	iTRAQ	1.00	1.15	0.84	0.88	0.76	1.01	1.09	1.06	0.68
	MRM	1.00	1.59	0.78	0.47	0.55	1.22	0.58	1.38	
tr|F1RLJ4|F1RLJ4_PIG (SCAMP3)	iTRAQ	1.00	1.11	0.93	0.86	0.96	0.95	0.87	0.96	0.65
	MRM	1.00	1.56	1.46	0.74	0.90	0.99	1.00	1.15	

### Functional annotation of DAPs

**Fig. 5:**
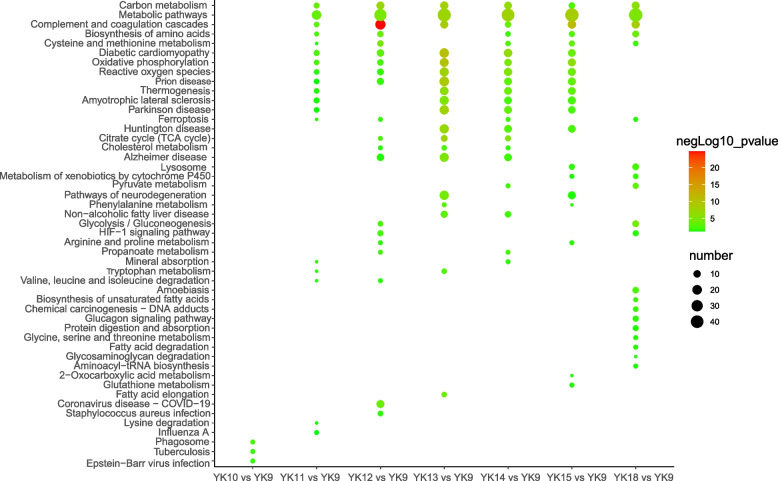
KEGG analysis of different abundance proteins. Color represents significance, and dot size represents the number of genes clustered in this KEGG term. Green shows significant difference. The number in the parentheses means the number of differentially abundance protein in this term. YK: Days of pregnancy for Yorkshire pigs. “number”: the number of different abundance proteins

To analyze the functions of DAPs in porcine endometrium during preimplantation, Gene Ontology (GO) and Kyoto Encyclopedia of Genes and Genomes (KEGG) analyses were carried out on the DAPs in 7 comparisons (YK10 vs. YK9, YK11 vs. YK9, YK12 vs. YK9, YK13 vs. YK9, YK14 vs. YK9, YK15 vs. YK9 and YK18 vs. YK9). Significantly enriched GO terms mainly included “serine − type endopeptidase inhibitor activity”, “extracellular space”, “extracellular region”, and “negative regulation of endopeptidase activity” in all comparisons (Additional file 3: Fig. S1). The KEGG analysis showed that the top 20 significantly enriched pathways in 7 comparisons were related to metabolism, including “Carbon metabolism”, “Cysteine and methionine metabolism” and “Citrate cycle (TCA cycle)” (Fig. 5 and Additional file 4: Table S3). Notably, “oxidative phosphorylation” was a significantly enriched pathway in the YK11 vs. YK9 YK12 vs. YK9, YK13 vs. YK9, YK14 vs. YK9 and YK15 vs. YK9 comparison groups (Fig. 5).

### The role of retinol binding protein 4 (RBP4) in endometrial epithelial cells (EECs) and endometrial stromal cells (ESCs)

**Fig. 6:**
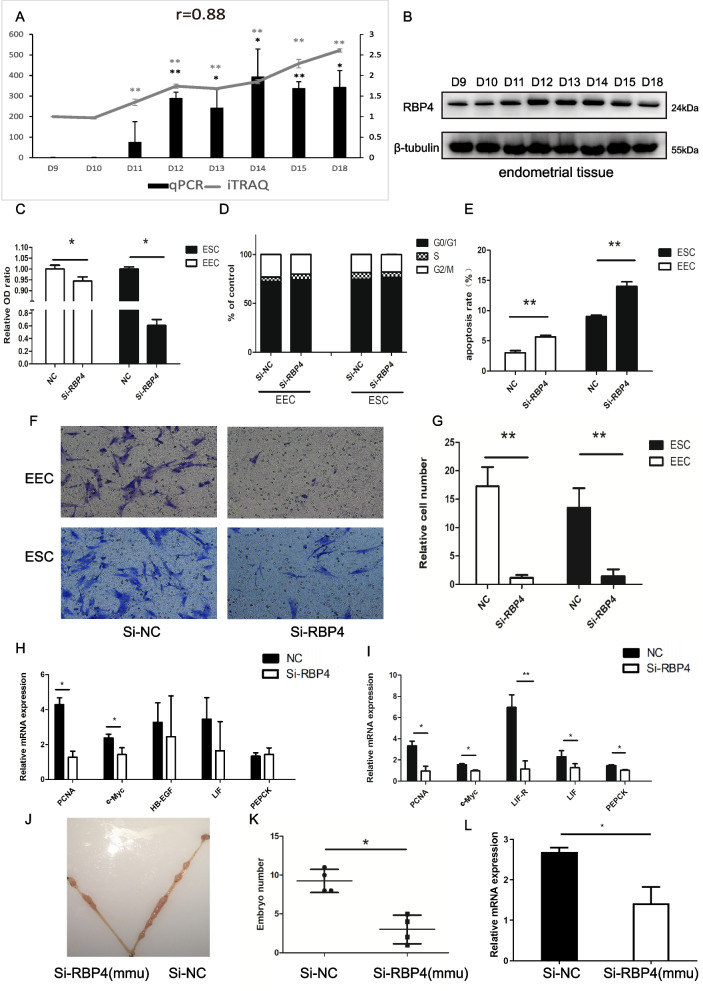
The role of RBP4 in EECs and ESCs. **A** Validation of iTRAQ results of RBP4 by using RT-qPCR. Gray lines and blank column representing the values were determined by iTRAQ and RT-qPCR, respectively. The *r-value* shows Pearson’s correlation between two sets of values determined by above two methods. **B** The results of Western blot. **C** Results of a CCK8 assay after treatment with Si-RBP4 during EECs proliferation. **D**, **E** The effects of Si-RBP4 on cell cycle and apoptosis. **F**, **G** After treatment with Si-RBP4, cell migration was determined using a transwell assay. In H, relative cell number represents the average cell number found. **H**, **I** The effect of Si-RBP4 on the mRNA expression levels of genes related to embryo implantation. **J**, **K** Comparison of the number of implantation sites between Si-RBP4 (mmu) injected uterine horn (left, L) and NC-injected uterine horn (right, R) (*n* = 5). **L** The expression of RBP4 is presented as a value relative to β-actin (*n* = 3). Results are mean ± s.d. *P < 0.05, **P < 0.01 (Student’s t-test). Scale bar: 200 µm. NC, negative control

In the endometrial tissues of pigs, the mRNA and protein expression levels of RBP4 were significantly upregulated on D12, D13, D14, D15 and D18 compared with D9 (Fig. 6A). The Pearson correlation coefficient between the real-time quantitative PCR (RT‒qPCR) value and iTRAQ value of RBP4 was 0.88 (Fig. 6A). The Western blot of RBP4 is shown in Fig. 6B (Additional file 5: Fig. S2). Previous studies have also indicated that retinol binding protein acts as a chaperone to transport vitamin A to the conceptus and that RBP4 plays a vital role in cell function [6, 19], so we investigated the role of RBP4 in EECs and ESCs. Si-RBP4 (which acts as an inhibitor of RBP4) was transfected into EECs and ESCs. The mRNA expression level of RBP4 was decreased after the transfection of Si-RBP4 in EECs and ESCs (*P* < 0.05) (Additional file 6: Fig. S3). The Cell Counting Kit-8 (CCK-8) results showed that knockdown of RBP4 significantly inhibited the proliferation of EECs and ESCs (*P* < 0.05) (Fig. 6C). The cell cycle analysis and apoptosis analysis showed that Si-RBP4 induced G0/G1 phase block and apoptosis of EECs and ESCs (*P* < 0.01) (Fig. 6D, Fig. 6E and Additional file 7: Fig. S4). Furthermore, Si-RBP4 remarkably hindered the migration of EECs and ESCs (*P* < 0.01) (Fig. 6F and Fig. 6G). Altogether, knockdown of RBP4 inhibited the proliferation and migration of EECs and induced their G0/G1 phase block and apoptosis.

To analyze the regulatory mechanism of RBP4 in EECs and ESCs, we detected changes in the mRNA expression levels of genes related to embryo implantation after transfection with Si-RBP4. We observed the downregulation of proliferating cell nuclear antigen (PCNA) and transcriptional regulator Myc-like (c-Myc) expression at the mRNA level in EECs and ESCs (*P* < 0.05) and the downregulation of the mRNA expression levels of LIF receptor subunit alpha (LIFR) (*P* < 0.01), LIF interleukin 6 family cytokine (LIF) and phosphoenolpyruvate carboxykinase 1 P-PEPCK in ESCs (*P* < 0.05) (Fig. 6H and Fig. 6I). Moreover, loss of function of RBP4 in vivo resulted in a decreased number of implanted mouse embryos (Fig. 6J and Fig. 6K). The expression of RBP4 in the uterine horn injected with siRBP4 was lower than that in the control uterine horn (*P* < 0.05) (Fig. 6L).

## Discussion

The present study aimed to explore the temporal protein abundance patterns in porcine endometrium during early pregnancy and identify vital proteins that may regulate embryo implantation. Comprehensive profiling of the expression of protein in endometrial tissues from pregnant gilts (D9, D10, D11, D12, D13, D14, D15 and D18) was performed via iTRAQ technology. 2DE is a top-down approach that quantifies the differentially abundant proteins at the protein level before identifying the protein by LC–MS/MS, while the iTRAQ method is a bottom-up approach in which the whole proteome is first digested with trypsin; then, the generated peptides are separated by chromatography and identified and measured by mass spectrometry [20, 21].

We sequenced endometrial tissue from these eight periods. Endometrial tissue was obtained from the distal, proximal, and middle parts of the uterine horn according to previously reported methods. Some studies also chose the site of hyperemic zones because they selected the 14th day of gestation, which is easy to observe (observation is not easy earlier) [22, 23]. To ensure the consistency of sample sites, we used the same site of sample tissue. However, the endometrial samples were consistent with previous studies, which included the lamina epithelialis, lamina propria, and tela submucosa but not the tunica muscularis. However, recent research has shown that the published laser microdissection technique can separate layers of endometrial cell types [24–26]. On the other hand, we used whole endometrial fragments, which may have resulted in proteome profiling.

We also identified candidate DAPs that may have a crucial effect on embryo implantation. Some studies were published that focused on the investigation of the gene expression profiles in the porcine endometrium during the early gestation phases [12–15]. However, investigation of the protein abundance in the endometrium of pigs during the whole process from the beginning to the completion of embryo implantation is important for understanding the mechanism of uterine function. Among DAPs, we found that the number of downregulated proteins in all comparison groups was greater than that of upregulated proteins except YK10 vs. YK9. To further explore the abundance profile of DAPs in regulating embryo implantation, STEM analysis was performed for all DAPs. The results showed that two of three typical expression patterns of proteins were characterized by decreased levels from D9 to D18. Twelve and 15 days were the two key time points during the preimplantation phases.

Embryos begin to attach to the surface of the endometrium on D13 [6]. In our study, the abundance profiles of protein in the YK10 vs. YK9 and YK11 vs. YK9 comparison groups were not directly clustered with the other groups. We speculated that the protein expression profile of the endometrium changes after the embryo reaches the endometrium. The results also indicated that protein abundance in the endometrium changed significantly from Day 12 of pregnancy, which was consistent with previous studies. Another study focused on uterine luminal fluid proteins on D9 of the oestrus cycle and D9, 12 and 15 of pregnancy and observed significant changes between D9 and 12 of pregnancy [27].

Porcine endometrium undergoes functional changes for the success of embryo implantation [1, 28]. The GO term “binding” contributed to embryo implantation and was related to upregulated genes in Meishan pigs compared to Yorkshire pigs [12]. These GO terms (e.g., “unfolded protein binding”, “thyroid hormone binding” and “laminin binding”) involved in the remodeling of endometrium were enriched in all comparison groups (YK11 vs. YK9, YK12 vs. YK9, YK13 vs. YK9, YK14 vs. YK9, YK15 vs. YK9 and YK18 vs. YK9) [29–31]. Many previous studies have demonstrated that changes in maternal immunity are beneficial for the establishment of pregnancy during the embryo implantation period, which participates in the inflammatory response [32–35]. We further classified the significantly enriched GO terms according to molecular function, cellular component and biological process. “Serine-type endopeptidase inhibitor activity” was the only molecular function GO term that was enriched in all comparison groups. “Extracellular region” and “extracellular space” were enriched cellular component GO terms in all comparison groups, and other studies have found that this signaling pathway was also enriched at D14 of pregnancy [22, 36]. The genes coding for extracellular proteins have cell adhesion and immune functions. Extracellular matrix remodeling is necessary for ectopic endometrium implantation. Additionally, “negative regulation of endopeptidase activity” was enriched in all comparison groups and was related to biological processes.

Here, many pathways (e.g., “Carbon metabolism” and “Cysteine and methionine metabolism”) related to metabolism were enriched from D11 to 18. Moreover, “oxidative phosphorylation” and “citrate cycle (TCA cycle)” were enriched in five comparison groups (YK11 vs. YK9, YK12 vs. YK9, YK13 vs. YK9, YK14 vs. YK9, YK15 vs. YK9), which focused on the day of implantation. We hypothesized that the energy supply was necessary after the initiation of implantation. In addition to the change in the endometrial signaling pathway, there was also an influence of the number of conceptuses [37]. Variations within the number of conceptuses result in variable levels of their signals in the uterine lumen; therefore, the maternal response could be different, which is also related to the number of corpora lutea. Variation in the number of corpora lutea may result in different levels of P4 affecting the endometrium [37].

According to previous reports on differentially abundant proteins, we found that many proteins regulating embryo implantation were differentially abundant in our results. β-catenin and DPP4 are associated with embryonic development [38, 39]. Compared with previous transcriptome sequencing studies, we found that the protein levels of some key genes (STAT1, ISG15, AKR1B1 and ANXA8) regulating embryo attachment were also differentially abundant [40, 41]. In addition, some hormone-regulating proteins are differentially abundant, such as ER and GPX3 [42].

RBP4, a key factor related to placentation, was abundant in the luminal epithelium [43]. A previous study also showed that the mRNA expression level of RBP4 was upregulated on D12 or D15 compared with D9 via RNA-seq and RT‒qPCR [12]. Here, we found that the expression level of RBP4 in pigs from D11 to D18 was significantly higher than that on D9. A previous study indicated that endometrial remodeling, including cell proliferation, apoptosis, cell migration and cytoskeleton organization, imposes strong effects on embryo implantation [44]. We identified that knockdown of RBP4 inhibited proliferation and migration but induced apoptosis of EECs and ESCs. The knockdown of RBP4 suppressed the mRNA expression level of c-Myc involved in cell proliferation, migration and apoptosis [45–47]. Furthermore, this study verified that loss in function of RBP4 in vivo could decrease the number of implanted embryos. The above data indicate that RBP4 could regulate the function of EECs and ESCs to influence endometrial receptivity.

In conclusion, the temporal expression of proteins in the endometrium during embryo implantation was profiled. Our research provides a comprehensive overview of the establishment process of endometrial receptivity during early pregnancy in pigs. Furthermore, we found that RBP4 may exert a significant impact on the process of embryo implantation by regulating the proliferation, apoptosis and migration of EECs and ESCs.

## Materials and methods

### Animals and tissues

In this study, twenty-four YK gilts, which were similar in age and genetic background, were randomly assigned to eight different pregnancy groups. Furthermore, all the gilts mated with the same boar in the third oestrus cycle with the artificial insemination method. Uteri were collected from the gilts that were subsequently slaughtered on D9 (*n* = 3), 10 (*n* = 3), 11 (*n* = 3), 12 (*n* = 3), 13 (*n* = 3), 14 (*n* = 3), 15 (*n* = 3) and 18 (*n* = 3) of gestation. Then, the uteri were flushed with PBS quickly and subsequently opened longitudinally along the anti-mesometrial side. The endometrial tissues of eight periods were collected from three locations of each uterine horn: proximal, medial, and distal. The tissue samples were frozen in liquid nitrogen rapidly and stored at -80 °C for protein extraction.

### Protein extraction

For iTRAQ analysis, the twenty-four samples were homogenized (0.1 g) using a ceramic mortar and pestle and precooled with liquid nitrogen. Then, tissues were disrupted in lysis buffer (with enzyme inhibitors) by a TissueLyser machine. The mixtures were centrifuged at 25,000 × g for fifteen minutes. The supernatant was removed and mixed with 1 volume of cold acetone and stored at -20 °C for two hours. Subsequently, the samples were centrifuged at 25,000 × g for fifteen minutes, and the supernatant was removed again. Lysis buffer was added to dissolve the pellets. To reduce the disulfide bond of peptides, 10 mM DTT was added to the solution and maintained at 56 °C for one hour. A total of 55 mM IAM was added to the solution, which was maintained in a dark room for forty-five minutes. One volume of chilled acetone was added to the solution, stored at -20 °C for two hours and then centrifuged at 25,000 × g for fifteen minutes. Then, the solution was removed. Finally, lysis buffer was added to dissolve the pellet to obtain the protein solution.

### Protein digestion and iTRAQ labeling

One hundred micrograms of protein from each sample was transferred to a 1.5 ml new tube for digestion. Then, each sample was mixed with 5 μg of Trypsin Gold for four hours at 37 °C. Subsequently, 5 μg of Trypsin Gold was added for digestion at 37 °C for eight hours. After trypsin digestion, the peptides were dried by vacuum centrifugation and redissolved in 0.5 M TEAB. Finally, the iTRAQ labeling of prepared peptide samples was performed according to the iTRAQ Reagent 8-plex Kit manufacturer’s protocol. The samples were labeled YK9-113, YK10-114, YK11-115, YK12-116, YK13-117, YK14-118, YK15-119, and YK18-121.

### Peptide fractionation and LC‒ESI‒MS/MS analysis

The iTRAQ-labeled peptides were fractionated by RP chromatography using the Shimadzu LC-20AB HPLC Pump system. Each fraction was resuspended in buffer A (5% CAN, 0.1% FA). Centrifuging the mixture at 20,000 × g for ten minutes, we obtained the supernatant, of which the peptide final concentration was approximately 0.5 g/l. Finally, according to the experimental protocol, we can acquire the results. The data were acquired with a 2.5 kV ion spray voltage, 30 psi curtain gas, 15 psi nebulizer gas and interface heater temperature of 150 °C. In addition, the data were obtained with a Triple TOF5600 System (AB SCIEX, Concord, ON) fitted with a Nanospray III source (AB SCIEX, Concord, ON) and a pulled quartz tip as the emitter (New Objectives, Woburn, MA) and controlled with Analyst 1.6 software (AB SCIEX, Concord, ON).

### Bioinformatics analysis

The proteins were identified using the UniProt Pig database (http://www.uniprot.org). The raw MS/MS data were converted into MGF format by the ProteoWizard tool msConvert, and the exported MGF files were searched using Mascot version 2.3.02 in this project against the selected database. The proteins were quantified using automated software called IQuant. In this study, functional annotation of differentially abundant proteins was conducted on the basis of DAVID (https://david.ncifcrf.gov/). The GO terms were obtained by ticking the “GOTERM_BP_DIRECT”, “GOTERM_CC_DIRECT” and “GOTERM_MF_DIRECT” of “Gene Ontology”. The KEGG pathways were obtained by ticking the “KEGG_PATHWAY” of “Pathways” (www.kegg.jp/kegg/kegg1.html) [48–50]. These pathways and GO terms with *P* value < 0.05 were considered significantly enriched pathways and GO terms.

The profiles of differentially abundant proteins over time were visualized by Short Time-series Expression Miner v 1.3.8 (STEM, http://www.cs.cmu.edu/~jernst/stem/). The STEM clustering method was selected as the clustering method, and other parameters were set as default. The protein abundance profiles were ordered by significance.

The fold change values of comparisons were normalized by using the log2 (value). HCA was conducted using imageGP (http://www.ehbio.com/ImageGP/index.php/Home/Index/PHeatmap.html).

### MRM

#### Protein extraction

Samples were ground into powder in liquid nitrogen and extracted with lysis buffer (7 M urea, 2 M thiourea, 4% CHAPS, 40 mM Tris–HCl, pH 8.5) containing 1 mM PMSF and 2 mM EDTA (final concentration). After 5 min, 10 mM DTT (final concentration) was added to the samples. The suspension was sonicated at 200 W for 15 min and then centrifuged at 4 °C and 30 000 × g for 15 min. The supernatant was mixed well with a 5 × volume of chilled acetone containing 10% (v/v) TCA and incubated at -20 °C overnight. After centrifugation at 4 °C and 30 000 g, the supernatant was discarded. The precipitate was washed with chilled acetone three times. The pellet was air-dried and dissolved in lysis buffer (7 M urea, 2 M thiourea, 4% NP40, 20 mM Tris–HCl, pH 8.0–8.5) [51, 52].

The suspension was sonicated at 200 W for 15 min and centrifuged at 4 °C and 300,000 × g for 15 min. The supernatant was transferred to another tube. To reduce disulfide bonds in proteins of the supernatant, 10 mM DTT (final concentration) was added and incubated at 56 °C for 1 h. Subsequently, 55 mM IAM (final concentration) was added to block the cysteines and incubated for 1 h in the darkroom. The supernatant was mixed well with a 5 × volume of chilled acetone for 2 h at -20 °C to precipitate proteins. After centrifugation at 4 °C and 30 000 g, the supernatant was discarded, and the pellet was air-dried for 5 min, dissolved in 500 μL 0.5 M TEAB (Applied Biosystems, Milan, Italy) and sonicated at 200 W for 15 min. Finally, samples were centrifuged at 4 °C and 30 000 × g for 15 min [51, 52]. The supernatant was transferred to a new tube and quantified using a Bradford kit (Bio-Rad). The proteins in the supernatant were stored at -80 °C for further analysis.

#### Protein digestion

Total protein (100 μg) was removed from each sample solution, and then the protein was digested with Trypsin Gold (Promega, Madison, WI, USA) at a protein:trypsin ratio of 30:1 at 37 °C for 16 h. After trypsin digestion, peptides were dried by vacuum centrifugation. Peptides were reconstituted in 0.5 M TEAB.

#### LC- MRM- MS

Samples were digested as described and spiked with 50 fmol of β-galactosidase for data normalization. MRM analyses were performed on a QTRAP 6500 mass spectrometer (SCIEX, Framingham, MA, USA) equipped with an LC-20AD nanoHPLC system (Shimadzu, Kyoto, Japan). The mobile phase consisted of solvent A, 0.1% aqueous formic acid, and solvent B, 98% acetonitrile with 0.1% formic acid. Peptides were separated on a C18 column (0.075 × 150 mm column, 3.6 μm) at 300 nL/min and eluted with a gradient of 5%-30% solvent B for 38 min, 30%-80% solvent B for 4 min, and maintenance at 80% for 8 min. For the QTRAP 6500 mass spectrometer, a spray voltage of 2400 V, nebulizer gas of 23 p.s.i., and a dwell time of 10 ms were used. Multiple MRM transitions were monitored using unit resolution in both Q1 and Q3 quadrupoles to maximize specificity [53].

### Cell preparation and culture conditions

Isolation and culture of porcine primary endometrial epithelial cells and endometrial stromal cells were performed according to previous research [12, 54]. The endometrium was separated and shredded with sterile scissors. After washing twice with PBS, the tissue pieces were incubated at 37 °C for 2.5 h with collagenase I (Gibco, NY, USA) and shaken vigorously every half hour. Undigested tissue pieces were removed by screen filtration. Then, the filtrate was centrifuged at 500 × g for 10 min to remove the supernatant (fraction rich in endometrial stromal cells). The pellet (epithelial‐rich fraction) was resuspended twice in PBS and recentrifuged (500 g, 10 min) twice. The supernatant (fraction rich in endometrial stromal cells) was recentrifuged (1000 g, 10 min) twice to obtain the pellet (stromal-rich fraction). The pellets of EECs and ESCs were suspended in Dulbecco’s modified Eagle’s medium/F-12 (DMEM/F12; 1:1) medium (Gibco) supplemented with 10% fetal bovine serum (Gibco) and 1% penicillin‒streptomycin (Gibco) and cultured in a 37 °C and 5% CO2 incubator [55]. The epithelial and stromal cells were then trypsinized with 0.25% trypsin–EDTA and placed in a cell culture flask (Corning, NY, USA) for subsequent experiments. Ninety percent purity of populations of endometrial cells was isolated.

### Transfection

All RNA oligonucleotides were designed and synthesized by GenePharm (Shanghai, China). EECs and ESCs were transfected with Si-RBP4 (sense: CCGGCUGAUCACUCACAAUTT, antisense: CCGGCUGAUCACUCACAAUTT) and Si-NC (sense: UUCUUCGAACGUGUCACGUTT, antisense: ACGUGACACGUUCGGAGAATT) using Lipofectamine® 2000 (Invitrogen Life Technology, Shanghai, China) according to the manufacturer’s instructions. The transfection efficiency was more than 90%.

### RNA extraction and real-time quantitative PCR (RT‒qPCR)

RNA was extracted from cells using TRIzol (Invitrogen) as recommended by the manufacturer, and the concentration and quality were measured by a NanoDrop 2000 (ThermoFisher, Waltham, USA) [54]. Complementary DNA (cDNA) was synthesized with a reverse transcription kit (Takara, Tokyo, Japan). Then, Mix (Toyobo, Japan) and specific primers for every gene were used to perform RT‒qPCR on a Real-time System (Roche, Basel, Switzerland) (Additional file 8: Table S4). The expression levels of genes were normalized to ribosomal protein S20 (RPS20) to obtain the relative expression using the 2^–ΔΔCt^ method according to a previous study [12, 56].

### Western blotting

Total protein was isolated from the endometrium using SDS Lysis Buffer (P0013B, Beyotime Ltd. China). Protein content was measured using an enhanced BCA protein assay kit (P0010, Beyotime, Ltd. China). The sample was denatured by heating, separated by SDS‒PAGE and transferred to a PVDF (polyvinylidene fluoride) membrane. Next, the membranes were blocked with 5% skimmed milk powder and separately probed with rabbit anti-RBP4 (catalog number: ab233138, Abcam) overnight at 4 °C with a final dilution of 1:1000 (v/v) in 5% milk. After three washes, the membranes were incubated with goat anti-rabbit secondary antibodies (GB23303, Sevicebio, Wuhan, China) at a 1:2000 dilution (v/v) at 37 °C for 1.5 h. Images of membranes treated with ECL (enhanced chemiluminescence) were captured by a Western blotting Detection System (Tiangen, Beijing, China).

### Cell proliferation assay

CCK-8 (Dojindo, Shanghai, China) was used to measure cell proliferation 48 h after transfection with the interference fragment of RBP4 following the manufacturer’s instructions [57, 58]. The optical density (OD) at 450 nm of each well plate was determined using a microplate reader (Bio-Rad, CA, USA).

### Cell apoptosis and cell cycle

Cell apoptosis analysis was carried out using the AnnexinV-FITC/PI apoptosis kit (Dojindo, Shanghai, China) according to the manufacturer’s protocol. Analyses were performed using a flow cytometer (Beckman, CA, USA).

For cell cycle analysis, the cells were separated by trypsin digestion. The precipitate was collected by centrifugation, washed twice, and then fixed. The supernatant was removed by centrifugation. Each sample was stained with propidium iodide (PI; KeyGEN BioTECH, Jiangsu) for 30 min in the dark at room temperature. Analyses were performed using a flow cytometer (Beckman, CA, USA).

### Cell migration assay

Cell migration was performed based on our previous study and assessed by a transwell assay that had 12 mm polycarbonate membranes with an 8.0 μm pore size (Corning, NY, USA) [54]. The EECs were resuspended in serum-free medium as a single-cell solution at 4 h posttransfection. Approximately 2 × 10^5^ EECs or ESCs were seeded on the upper chambers, and complete medium with 10% FBS was added to the lower chamber as a chemoattractant. After 24 h of incubation at 37 °C with 5% CO2, cells that migrated to the lower chamber were fixed with 4% paraformaldehyde for 5 min, stained with 0.1% crystal violet for 5 min, rinsed three times in PBS and subjected to an OLYMPUS DP80 microscope (Tokyo, Japan). The cell numbers of migration were obtained by counting five fields per membrane and represent the average of three independent experiments [54].

### Intrauterine injection of mice

It is difficult to perform in vivo experiments in pigs, so we used a mouse implantation model based on other studies and focused on pig or human implantation [12, 54, 59]. The date of finding the vaginal suppository after mating was designated as the first day. Intrauterine injection surgery under general anesthesia was performed on eight mice on Day 3 of pregnancy in the evening according to previous studies (Sun et al., 2014; Wang et al., 2019). Ten microliters of 10 μM mol/L Si-RBP4 (mmu) (sense: GGCCCUCCAGAAACAGUUUTT, antisense: AAACUGUUUCUGGAGGGCCTT) and inhibitor negative control (NC) were injected into the left and right uterine horns, respectively. Then, the wounds were sutured, and the mice were placed under a 37 °C warmer until the anesthesia diminished. To reduce the pain that the mice endured, temgesic was injected into the mice at 12, 24, and 48 h after surgery. On D5, the endometrium of three mice was isolated to examine the protein expression level of RBP4. On Day 7, five mice were killed, and their uteri were isolated to record the number of implanted embryos.

### Statistical analysis

Data were expressed as means ± standard deviation (SD) derived from at least three independent experiments. Student’s t-test was used for comparisons between two groups. One-way analysis of variance (ANOVA) test for multiple comparisons. *P* value < 0.05 was considered as statistically significant. * *P* < 0.05; ** *P* < 0.01.

## Supplementary Information

**Additional file 1.**

**Additional file 2.**

**Additional file 3.**

**Additional file 4.**

**Additional file 5.**

**Additional file 6.**

**Additional file 7.**

**Additional file 8.**

## Data Availability

All new sequencing data generated in this study have been deposited to ProteomeXchange Consortium via the PRIDE under accession number PXD030681.

## Abbreviations

ANOVA: Analysis of variance
2DE: Two-dimensional gel electrophoresis
CCK-8: Cell Counting Kit-8
cDNA: Complementary DNA
c-Myc: Transcriptional regulator Myc-like
DAPs: Differentially abundance proteins
EECs: Endometrial epithelial cells
ESCs: Endometrial stromal cells
GO: Gene Ontology
HCA: Hierarchical cluster analysis
iTRAQ: Isobaric tags for relative and absolutequantification
KEGG: Kyoto Encyclopedia of Genes and Genomes
LIF: LIF interleukin 6 family cytokine
LIFR: LIF receptor subunit alpha
NC: Negative control
OD: Optical density
P-PEPCK: Phosphoenolpyruvate carboxykinase 1
PCNA: Proliferating cell nuclear antigen
PVDF: Polyvinylidene fluoride
RBP4: Retinol binding protein 4
RPS20: Ribosomal protein S20
RT-qPCR: Real time quantitative PCR
SD: Standard deviation
STEM: Short time-series expression miner
YK: Yorkshire
