# Supplementary material for: Proteomic profiles and the function of RBP4 in endometrium during embryo implantation phases in pigs
Source: BMC Genomics. 2023 Apr 13;24:200. doi: 10.1186/s12864-023-09278-5 (PMC10099840; doi:10.1186/s12864-023-09278-5)
Supplement: Supplementary file 1 — Additional file 1. [file 12864_2023_9278_MOESM1_ESM.pdf]

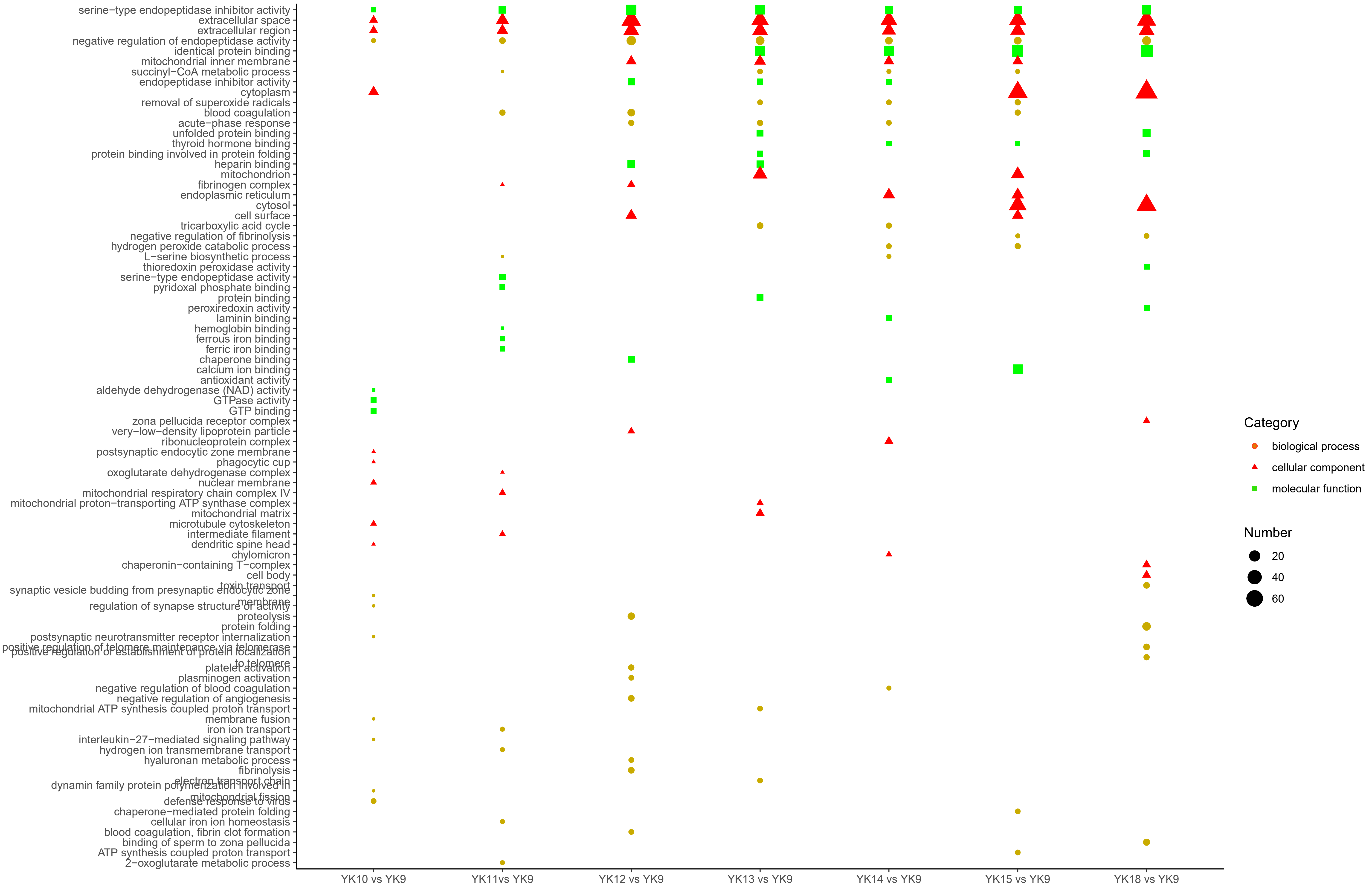

Figure S1. GO analysis of differentially abundance proteins. YK: Days of pregnancy for Yorkshire pigs. "number": the number of differentially abundance proteins.
