# Supplementary figures and images for: Proteomic profiles and the function of RBP4 in endometrium during embryo implantation phases in pigs
Source: BMC Genomics. 2023 Apr 13;24:200. doi: 10.1186/s12864-023-09278-5 (PMC10099840; doi:10.1186/s12864-023-09278-5)

RBP4

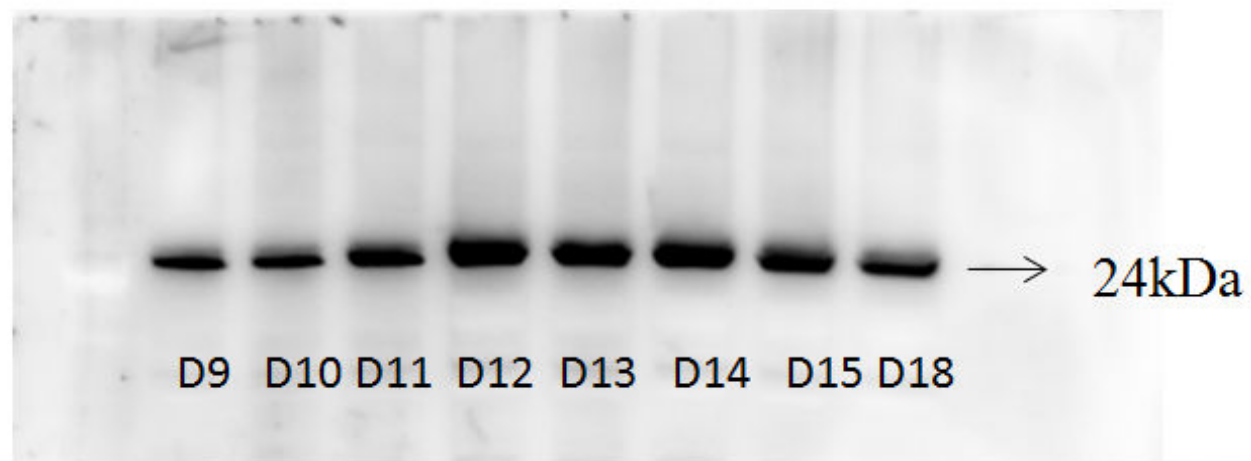

$\beta$ -tubulin

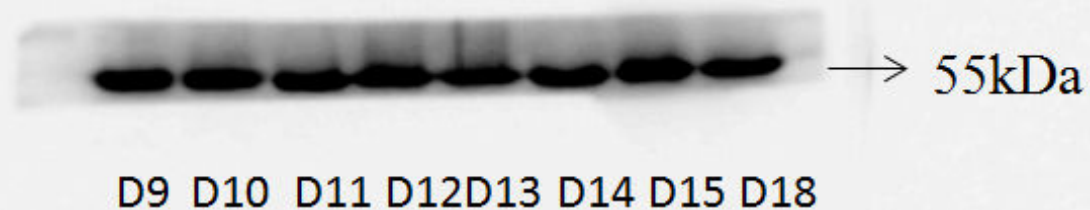

Figure S2. The Western Blot of RBP4 (24kDa) and TUBLIN (55kDa).

Supplement: Supplementary file 2 — Additional file 2. [file 12864_2023_9278_MOESM2_ESM.pdf]

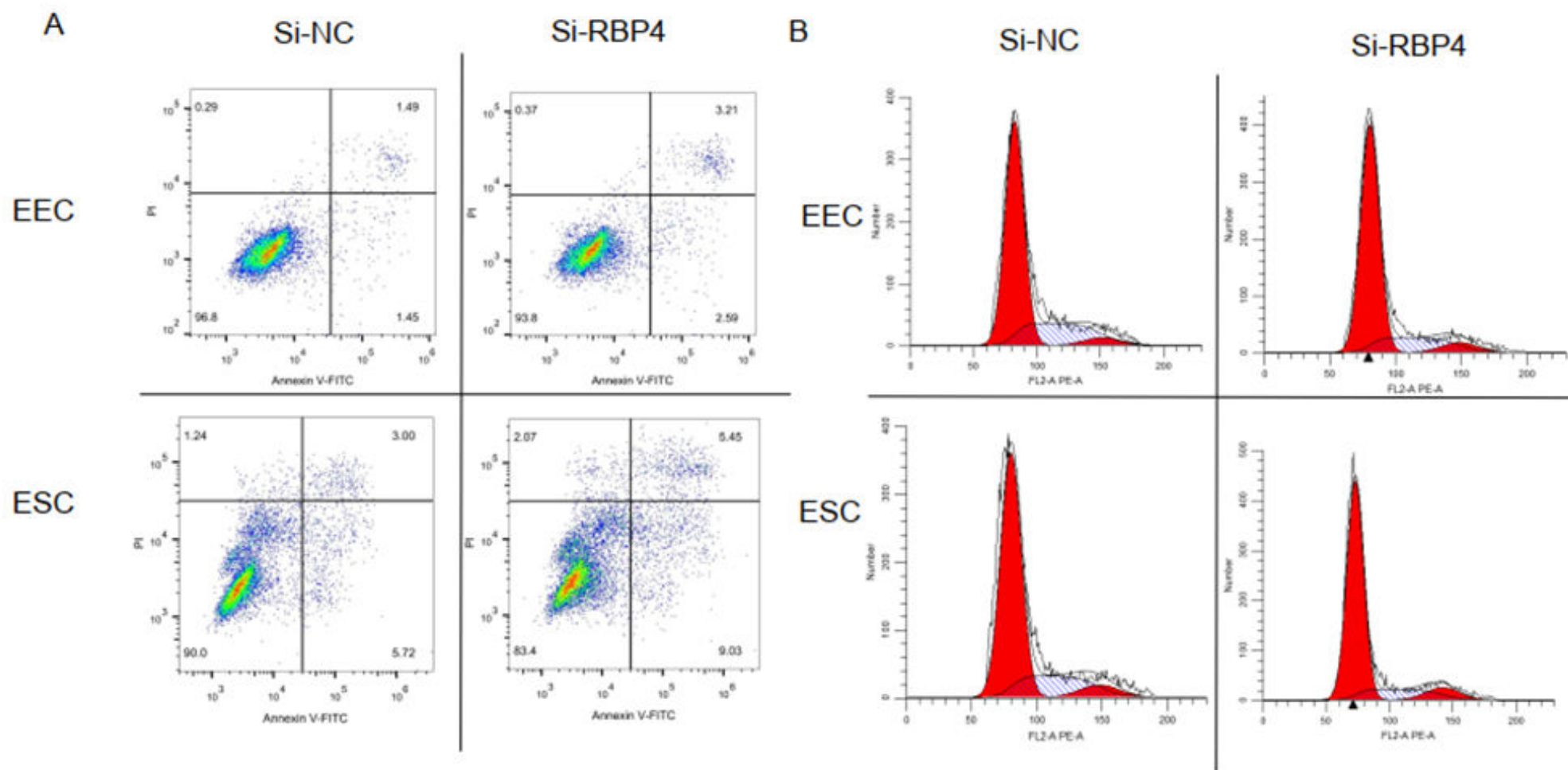

Figure S4: Flow cytometry of apoptosis (A) and cell cycle (B)

Supplement: Supplementary file 4 — Additional file 4. [file 12864_2023_9278_MOESM4_ESM.pdf]
