# Supplementary material for: Proteomic profiles and the function of RBP4 in endometrium during embryo implantation phases in pigs
Source: BMC Genomics. 2023 Apr 13;24:200. doi: 10.1186/s12864-023-09278-5 (PMC10099840; doi:10.1186/s12864-023-09278-5)
Supplement: Supplementary file 3 — Additional file 3. [file 12864_2023_9278_MOESM3_ESM.pdf]

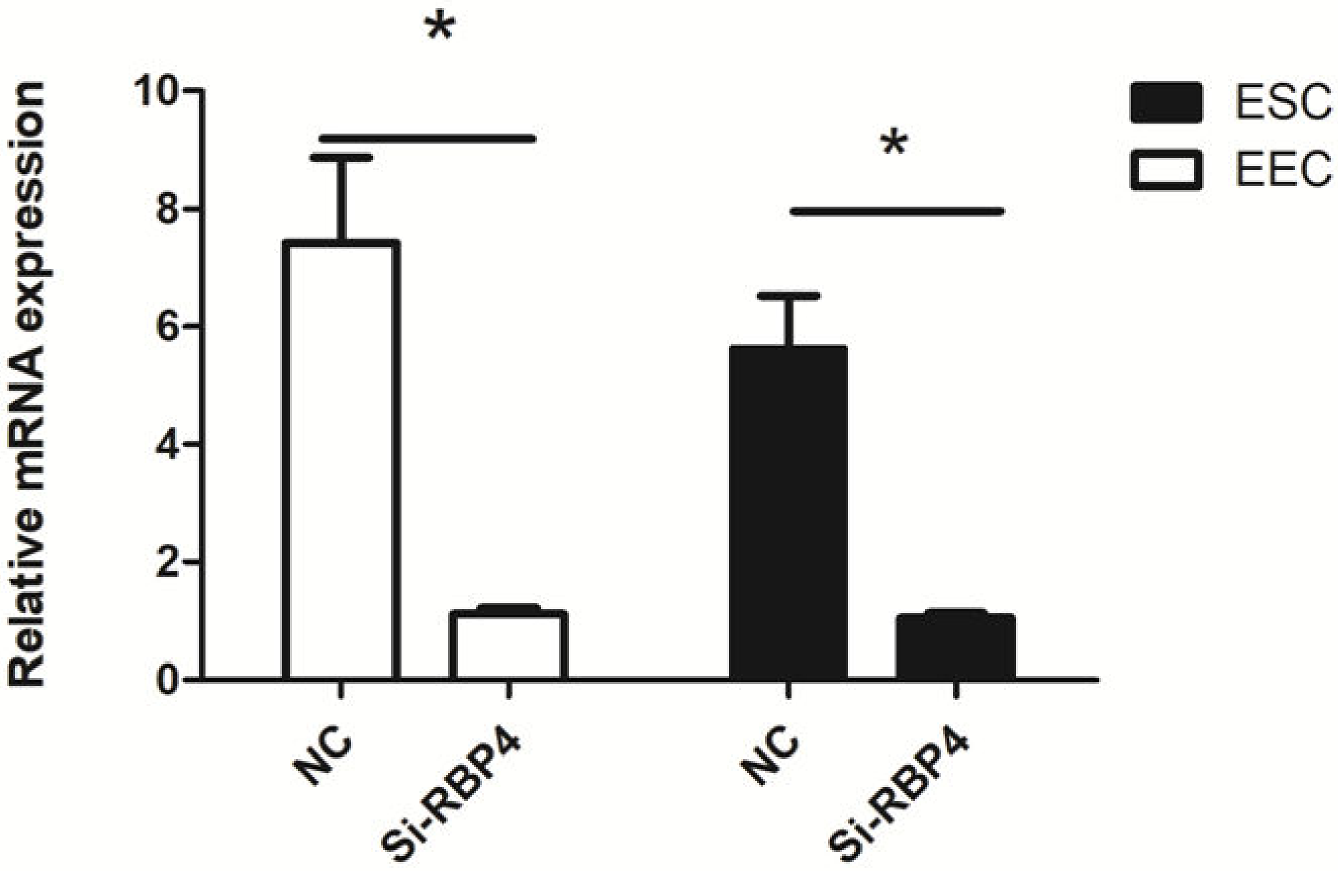

Figure S3. **The expression level of RBP4 after treatment with Si-RBP4 in EECs and ESCs.** RBP4 levels are presented relative to RPS20 levels. Results are mean ± s.d. $^*P<0.05$ (Student's t-test). NC, negative control.
